# Palmare Papeln – Ein Warnzeichen

**DOI:** 10.1007/s00105-022-05032-2

**Published:** 2022-07-12

**Authors:** Lukas Koch, Karin Plaschg, Emina Talakic, Martin H. Stradner

**Affiliations:** 1 Universitätsklinik für Dermatologie und Venerologie, Medizinische Universität Graz, Auenbruggerplatz 8, 8036 Graz, Österreich; 2 Klinische Abteilung für Allgemeine Radiologische Diagnostik, Medizinische Universität Graz, Graz, Österreich; 3 Klinische Abteilung für Rheumatologie und Immunologie, Medizinische Universität Graz, Graz, Österreich

## Anamnese und klinische Untersuchung

**Figure 1:**
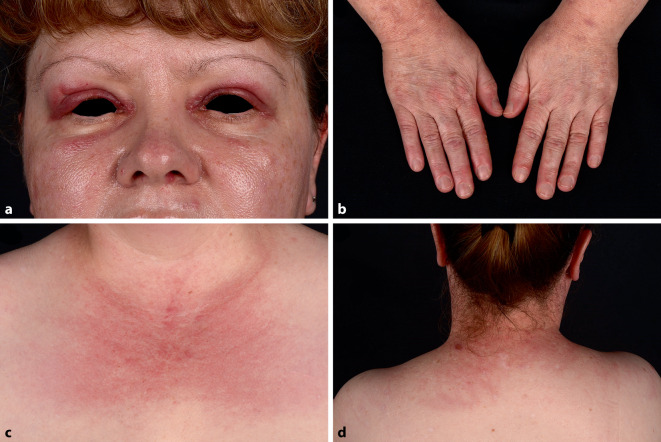

**Figure 2:**
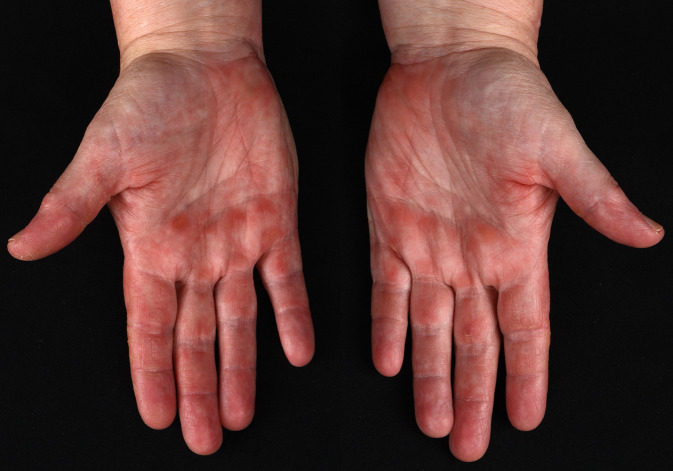

Eine 49-jährige österreichische Frau berichtet über zunehmende Hautveränderungen seit 3 Monaten ohne Systemzeichen wie Fieber, Atemnot, Muskelschmerzen, Muskelschwäche oder Schluckbeschwerden. Die Hautuntersuchung zeigte periorbitale livide Erytheme mit Ödemen der Oberlider, eine livide Verfärbung der dorsalen Metakarpal- und Interphalangealgelenke sowie ein erythematös-livides Exanthem rund um den gesamten Hals (Abb. 1a–d). Auffallend waren zusätzlich erythematöse Papeln am Hypothenar als auch an den palmaren Metakarpal- und Interphalangealgelenken beidseits (Abb. 2).

## Diagnostische Untersuchungen

C‑reaktives Protein, Kreatinkinase, Aldolase, Myoglobin und Lactatdehydrogenase waren in den Normbereichen. ANA- und ENA-Titer waren normal, jedoch zeigten sich Antikörper gegen Anti-MDA5 („melanoma differentiation-associated gene 5“). Die histologische Untersuchung einer Stanzbiopsie des oberen Augenlides ergab eine Interface-Dermatitis. Ein Lungenröntgen und eine Lungenfunktion 4 Wochen vor Vorstellung waren unauffällig.

**Figure 3:**
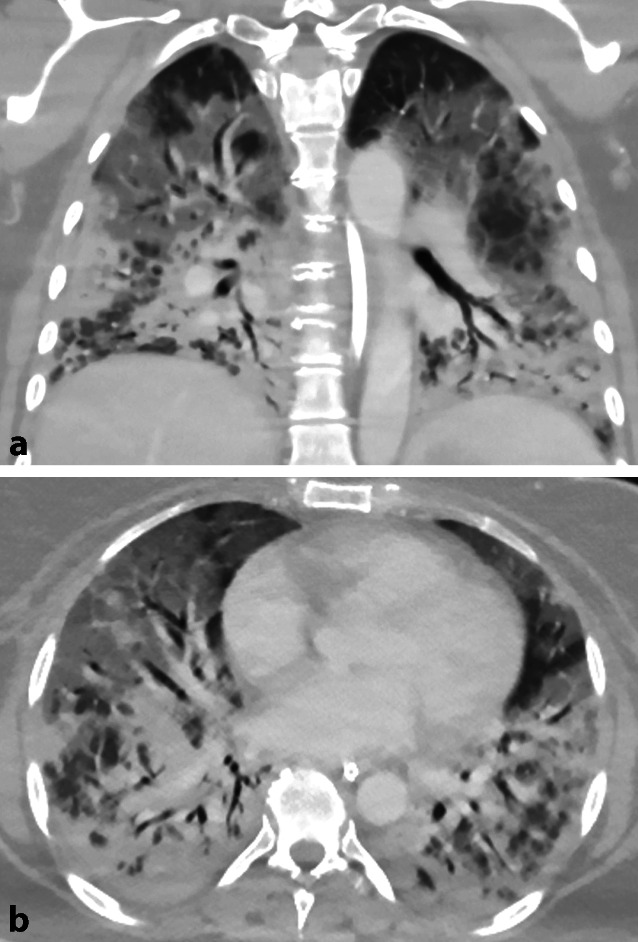

Nach 2 Monaten entwickelte die Patientin Gelenkschmerzen und eine zunehmende Atemnot. Eine daraufhin durchgeführte Computertomographie des Thorax zeigte Bronchiektasien, Milchglastrübungen und Konsolidierungsareale in beiden Lungenflügeln (Abb. 3).

## Wie lautet Ihre Diagnose?

## Diskussion

Die initiale Hautuntersuchung zeigte das typische Erscheinungsbild einer Dermatomyositis (DM) mit positivem „heliotrope sign“ (Heliotrop-Zeichen, eine livide Verfärbung der oberen Augenlider, nicht selten gemeinsam mit Lidödemen), „shawl sign“ (Schal-Zeichen, livide Makulae und/oder Teleangiektasien um den Hals) und „Gottron’s sign“ (Gottron-Zeichen, eine livide Verfärbung über Gelenken wie dorsalen Fingergelenken, Knien oder Ellbogen).

Die klinischen Symptome und Laboruntersuchungen gaben keinen Hinweis auf eine Muskelbeteiligung, weshalb die Diagnose amyopathische DM gestellt und für den Hautbefall Hydroxychloroquin 400 mg täglich eingeleitet wurde. Eine Schnittbildgebung des Thorax wurde empfohlen, jedoch von der Patientin nicht durchgeführt.

Zwei Monate später entwickelte die Patientin Gelenkschmerzen und eine zunehmende Atemnot. Mittels CT des Thorax wurde eine rapid-progressive interstitielle Lungenerkrankung diagnostiziert und eine Steroid-Puls-Therapie eingeleitet (1 g Prednisolon täglich für 3 Tage, gefolgt von 100 mg täglich). Bedauerlicherweise verschlechterte sich der Zustand der Patientin rasch, und sie entwickelte ein akutes Lungenversagen. Die darauffolgende intensivmedizinische Behandlung u. a. mit extrakorporaler Membranoxygenierung verkomplizierte sich durch extensive venöse Thrombosen. Nach einer Behandlung mit Heparin und einer Lyse mit Alteplase kam es zu einer Subarachnoidalblutung, an welcher die Patientin verstarb. Im Rahmen der Obduktion zeigte sich kein Hinweis auf ein Malignom.

Erythematöse palmare Papeln, welche histologisch mit einer okklusiven Vaskulopathie assoziiert sind, stellen ein typisches klinisches Zeichen für eine Anti-MDA5-Antikörper assoziierte Dermatomyositis dar. Weitere bei diesem Krankheitsbild beschriebene Hauterscheinungen sind Alopezie, Hyperkeratosen und Schuppung der lateralen Finger, Pannikulitis sowie orale und kutane Ulzera [1, 2]. Diese Hauterscheinungen sollten als Warnzeichen dienen, und deren Kenntnis kann eine frühe Diagnose und Therapie der Erkrankung ermöglichen.

Die Diagnose basiert auf der Bestimmung von Anti-MDA5-Antikörpern, welche bei ca. 10–20 % aller Dermatomyositisfälle nachgewiesen werden können. Wenngleich eine Anti-MDA5-Antikörper-assoziierte DM üblicherweise amyopathisch verläuft, ist sie eine ernsthafte Verlaufsform der DM mit einem beträchtlichen Mortalitätsrisiko bei Lungenbeteiligung [3]. Eine Interstitielle Lungenerkrankung (ILD) wurde in diesem Zusammenhang in bis zu 100 % der Patienten beschrieben [4, 5] mit einer rapid-progressiven ILD in 38,1 %, einer chronischen ILD in 33,3 % und einer asymptomatischen ILD in 28,6 % [4].

In einer Metaanalyse zeigte sich, dass die gepoolte Sensitivität und Spezifität positiver Anti-MDA5-Antikörper für eine rapid-progressive Lungenerkrankung 77 % bzw. 86 % betragen [5].

Aus diesem Grund ist bei Patienten mit Anti-MDA5-positiver DM ein Screening auf interstitielle Lungenerkrankungen unerlässlich. Empfohlene Untersuchungen sind eine hochauflösende Computertomographie des Thorax (HRCT) sowie eine Lungenfunktionsuntersuchung inklusive Kohlenmonoxiddiffusionskapazität. Sollten die Untersuchungen initial normal sein, werden regelmäßige Wiederholungen empfohlen. Zusätzlich wurde ein erhöhtes Serumferritin als Risikofaktor für die Entwicklung einer rapid-progressiven ILD und einer schlechten Prognose beschrieben [6].

**Diagnose:** Anti-MDA5-Antikörper-assoziierte amyopathische Dermatomyositis mit rapid-progressiver interstitieller Lungenerkrankung

Retrospektiv war die Monotherapie mit dem Antimalariamittel Hydroxychloroquin für die Behandlung unserer Patientin nicht ausreichend. Obwohl Hydroxychloroquin nützlich ist zur Therapie einer kutanen Dermatomyositis, ist diese Substanz nicht ausreichend für eine innere Organbeteiligung. Beim Auftreten einer ILD ist eine frühzeitige kombinierte immunsuppressive Therapie notwendig, um eine progressive Lungenbeteiligung zu verhindern (z. B. die Kombination aus einem Steroid, einem Calcineurininhibitor und Cyclophosphamid i.v.). Diesbezüglich wurden rezent mehrere Behandlungsalgorithmen publiziert [7, 8]. Wenngleich derzeit nicht zugelassen, könnten in Zukunft auch innovative Behandlungskonzepte, wie z. B. der Januskinaseinhibitor Tofacitinib, eine therapeutische Rolle spielen [9]. Berücksichtigt werden sollte jedoch, dass nicht jeder Patient mit Anti-MDA5-positiver DM einen schweren Verlauf erleidet [5, 10], was die initiale Therapieentscheidung beeinflusst und die Notwendigkeit regelmäßiger Lungenuntersuchungen aufzeigt.
